# Neuromuscular and Perceptual Responses to Sub-Maximal Eccentric Cycling

**DOI:** 10.3389/fphys.2019.00354

**Published:** 2019-03-28

**Authors:** Pierre Clos, Davy Laroche, Paul J. Stapley, Romuald Lepers

**Affiliations:** ^1^CAPS UMR1093, Institut National de la Santé et de la Recherche Médicale (INSERM), Université de Bourgogne-Franche Comté, Dijon, France; ^2^INSERM CIC 1432, Plateforme d’Investigation Technologique, University Hospital of Dijon, Dijon, France; ^3^Neural Control of Movement Group, Faculty of Science, Medicine and Health, School of Medicine, Illawarra Health and Medical Research Institute, University of Wollongong, Wollongong, NSW, Australia

**Keywords:** negative work, pedaling, perception, corticospinal, rehabilitation

## Abstract

**Objective:**

Eccentric (ECC) cycle-ergometers have recently become commercially-available, offering a novel method for rehabilitation training. Many studies have reported that ECC cycling enables the development of higher levels of muscular force at lower cardiorespiratory and metabolic loads, leading to greater force enhancements after a training period. However, fewer studies have focused on the specific perceptual and neuromuscular changes. As the two latter aspects are of major interest in clinical settings, this review aimed to present an overview of the current literature centered on the neuromuscular and perceptual responses to submaximal ECC cycling in comparison to concentric (CON) cycling.

**Design:**

Narrative review of the literature.

**Results:**

At a given mechanical workload, muscle activation is lower in ECC than in CON while the characteristics of the musculo-articular system (i.e., muscle-tendon unit, fascicle, and tendinous tissue length) are quite similar. At a given heart rate or oxygen consumption, ECC cycling training results in greater muscular hypertrophy and strength gains than CON cycling. On the contrary, CON cycling training seems to enhance more markers of muscle aerobic metabolism than ECC cycling performed at the same heart rate intensity. Data concerning perceptual responses, and neuromuscular mechanisms leading to a lower muscle activation (i.e., neural commands from cortex to muscular system) at a given mechanical workload are scarce.

**Conclusion:**

Even though ECC cycling appears to be a very useful tool for rehabilitation purposes the perceptual and neural commands from cortex to muscular system during exercise need to be further studied.

## Introduction

Traditional rehabilitation programs have mainly comprised aerobic submaximal exercise (e.g., walking on a treadmill, concentric ergocycles, etc.). Stationary cycling is often used as it enables activities to be performed at very low intensities, isolating the mode of muscle contraction to a purely concentric (CON) one. CON muscle actions are characterized by the shortening of muscles in order to generate a force, while eccentric (ECC) contractions are an active lengthening of the muscle (e.g., walking downhill). One limit of locomotor CON exercise in patients with cardio-respiratory diseases, for example chronic obstructive pulmonary disease (Vieira et al., 2011) or chronic heart failure (Ritter et al., 2016; Chasland et al., 2017), is that it causes dyspnea and excessive fatigue. Interestingly, ECC exercise allows the production of similar muscle force to CON at a lower oxygen consumption, metabolic load, or 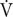
O_2_- (Abbott et al., 1952; Perrey et al., 2001) and perceived exertion (Besson et al., 2013). Moreover, it is now admitted that ECC training can be performed safely with limited muscle damage by increasing the exercise intensity very progressively and keeping it submaximal (LaStayo et al., 2013; Lovering and Brooks, 2013; Hoppeler, 2016). Consequently, the recent development of commercially available ECC cycle-ergometers has enabled new approaches to rehabilitation methods.

Published literature on ECC training has focused largely on cardiorespiratory, hemodynamic, and metabolic aspects. When exercising at a given mechanical workload, one bout of ECC cycling exercise induces lower increases in cardio-respiratory parameters (e.g., 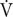
O_2_, breathing frequency, heart rate) compared to CON cycling (Abbott et al., 1952; Bigland-Ritchie and Woods, 1976; Hesser et al., 1977; Plante and Houston, 1984; Chung et al., 1999; Dufour et al., 2004; Laroche et al., 2013; Peñailillo et al., 2013, 2017a, 2018c; Lechauve et al., 2014; Chasland et al., 2017), as well as lower energy expenditure and glucose utilization but with greater fat utilization (Peñailillo et al., 2014). Moreover, at a given 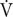
O_2_, an ECC bout cycling is known to elicit a higher heart rate (Hesser et al., 1977; Lipski et al., 2018). Regarding training effects, 8 weeks of ECC cycling did not improve 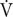
O_2peak_ while CON cycling at the same mechanical workload did (Besson et al., 2013; Lewis et al., 2018).

Recent studies have identified a lack of understanding of the neuromuscular (MacMillan et al., 2017) and perceptual (Pageaux et al., 2017) responses to ECC cycling. This is however, rarely the principal goal of applicable studies, but rather constitutes their secondary findings. Hence, the aim of this review was to gather and summarize the neuromuscular and perceptual responses to submaximal ECC in comparison to CON cycling.

## Methods

The search was carried-out in MEDLINE, Google Scholar, and Embase with filters set for articles written in English, using human studies and that were not reviews. The following search terms were used: “(*eccentric* OR *lengthening contraction* OR *negative work)* AND (*pedaling* OR *pedalling* OR *cycling).”* The search was restricted to papers from 1950 to October 1st 2018. Studies were included if they met the following criteria: (i) Reporting findings related to ECC cycling using the lower limbs, and (ii) Using protocols that consisted of submaximal exercise. Studies were excluded if they met the following exclusion criteria: (i) reporting only adaptations to exercises that were not neuromuscular or perceptual, and (ii) if the study did not compare results between ECC and CON cycling. The results were further divided into two subsections: (1) subjects’ responses during the exercise and resulting from one to two bouts of ECC cycling- deemed acute, and (2) subjects’ adaptations to several weeks of ECC training- deemed chronic. To increase the likelihood that all relevant studies were identified, electronic database searching was supplemented by examining the reference lists of all relevant retrieved articles.

## Results

**FIGURE 1:**
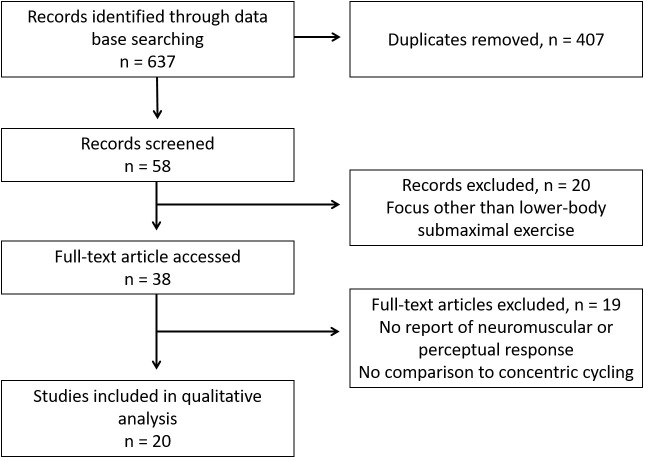
Flow diagram of the reviewing methods based on PRISMA guidelines (Liberati et al., 2009).

The literature search provided a total of 637 results including duplicates. Figure 1 details the flow followed to sort the articles. Fifty-seven articles from 1952 to October 1st 2018 included ECC cycling, among which 20 remained based on the inclusion and exclusion criteria (including 1 for which only an abstract was accessible). Seventeen out of the 20 remaining studies used recumbent cycle-ergometers. Unless specifically mentioned, the populations of the studies were constituted of healthy subjects, and results are written as mean ± SD unless stated.

### Acute Responses to ECC Cycling

Table 1 summarizes the neuromuscular and perceptual responses during and after one or two bouts or ECC cycling in comparison to CON cycling.

**Table 1:** Summary of the acute neuromuscular and perceptual responses during and after one or two bouts ECC cycling in comparison to CON cycling.

References	Sample	Methods	Main findings (in ECC compared to CON)
Bigland-Ritchie and Woods, 1976	2 trained subjects	50 rpm; 50–150 W Standard cycle-ergometer	- Lower *VL* integrated EMG
Chasland et al., 2017	11 chronic heart failure patients	40 rpm; 70% CON peak power Recumbent cycle-ergometer	- Similar muscle soreness, higher 24 and 48 h post
Dufour et al., 2004	8 healthy males	80 rpm; Incremental test (50 W +50 W/3 min) Recumbent cycle-ergometer	*At a given workload:* - Lower blood lactate
Elmer et al., 2010	18 recreational male cyclists	40 rpm; 5 min of one-leg ECC cycling at 40% of maximal CON power; Idem in CON with the contralateral leg; RPE measured during submaximal CON cycling, muscle pain during a squat Recumbent cycle-ergometer	- Similar perceived effort and muscle soreness, both higher 24 and 48 h post
Lechauve et al., 2014	8 healthy males	60 rpm; Incremental test (40 W + 40 W/2 min) Recumbent cycle-ergometer	*At a given workload:* - Lower *VL* integrated EMG - Similar *BB* integrated EMG *At a given* 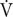 *O_2_:* - Similar *VL* EMG - Higher *BB* integrated EMG
Peñailillo et al., 2013	10 healthy men	1 bout of CON, 2 bouts of ECC (ECC1 and ECC2) 60 rpm, 30 min 60% CON peak power Recumbent cycle-ergometer	- Lower blood lactate, EMG, and perceived effort in ECC 1, but higher muscle pain - Larger decrease in MVC, SJ, and CMJ post-ECC 1 - No difference in increase in CK - Blood lactate was lower in ECC2 than ECC1 - No decrease in MVC, SJ, and CMJ post-ECC2 - No difference in perceived effort between ECC1 and ECC2
Peñailillo et al., 2015b	10 healthy males	1 bout of CON, 2 bouts of ECC (ECC1 and ECC2) 60 rpm, 30 min; 60% peak CON power Recumbent cycle-ergometer	- Larger ↘ in MVIC post-ECC but same RFD, and peak RFD post-exercise - RFD at 100–200 ms interval ↘ after ECC1 and immediately after ECC2, but did not change after CON
Peñailillo et al., 2017a	11 healthy males	60 rpm 65% peak CON power Recumbent cycle-ergometer	- *VL, VM, RF*, and *BF* integrated EMG were smaller
Peñailillo et al., 2018b	10 healthy males	60 rpm, 5 min of CON or ECC at 30, 60, 80, and 100% maximal CON power Recumbent cycle-ergometer	- Perceived effort and exertions showed distinct kinetics - Lower perception of effort and exertion in ECC at a given mechanical work rate
Perrey et al., 2001	6 healthy males	60 rpm 6 min bouts, 3 CON workloads (steady 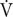 O_2_, 90% of VT, VT + 0.7 ( 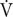 O_2peak_ + VT)), 1 ECC workload at the power of the highest CON load Standard cycle-ergometer	- Lower perceived exertion - Lower rectus femoris and vastus lateralis EMG *At a given 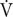 O_2_:* - Higher perceived exertion
Rakobowchuk et al., 2018	12 healthy males	30 rpm, 45 min 54% peak heart rate at 5 min of exercise Recumbent cycle-ergometer	*At a similar* 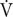 *O_2_* - *No difference in blood lactate*
Ward et al., 2018	11 healthy males	60 rpm, 4 min per load 3 submaximal workloads Upright cycle-ergometer	- Lower blood lactate - Lower muscle soreness immediately post-ECC, but larger 24 and 48 h after
Wells et al., 1986	3 healthy male students	Estimation of the internal mechanical work rate via cinematography at 30, 60, 90 rpm and 4 resistances Standard cycle-ergometer	- Similar increase in internal work with cadence

*ECC, eccentric; CON, concentric; KE, knee extensors; MVC, maximal voluntary isometric contraction; MVIC, maximal voluntary isometric contraction; VL, vastus lateralis; VM, vastus medialis, RF, rectus femoris; BF, biceps femoris; EMG, electromyography; RM, maximal repetition; RFD, rate of force development; rpm, revolution per minute; W, watts.*

#### Muscle Activation

At a given mechanical workload, the activation of the main muscles involved in pedaling (such as the vastus lateralis) during ECC was approximately half that of CON cycling (Bigland-Ritchie and Woods, 1976; Perrey et al., 2001; Peñailillo et al., 2013, 2017a; Lechauve et al., 2014). In contrast, at a given metabolic load, Lechauve et al. (2014) found no difference between the two contraction regimes. This study also described the electromyographic activity (EMG) of upper body muscles during the two activities, and reported three-times more important integrated EMG from the biceps brachii muscle during ECC cycling compared to CON cycling at the same metabolic load, even though this muscle was not directly involved in the pedaling action.

#### Biomechanical Features

At a given mechanical workload, muscle-tendon unit, fascicle, and tendinous tissue length (Peñailillo et al., 2017a), did not differ between ECC and CON. A similar increase in internal work with cadence was found in the two contraction regimes (Wells et al., 1986). Negative and positive peak crank torques within pedaling cycles were greater in ECC compared to CON cycling (32 and 48%, respectively) at the same average torque (Peñailillo et al., 2015a).

#### Muscle Metabolism

At a given mechanical workload, blood lactate was reported to be around 50% lower in ECC than in CON cycling (Plante and Houston, 1984; Dufour et al., 2004; Ward et al., 2018), and similar in the two contraction modes when pedaling at the same heart rate (Rakobowchuk et al., 2018). At a given mechanical workload, near-infrared spectroscopy measures showed a similar decrease of total myoglobin in ECC and CON while tissue oxygenation index was 16% greater in ECC cycling (Peñailillo et al., 2017a).

#### Neuromuscular Performance

At the same mechanical workload, post-exercise force, measured by maximal voluntary isometric contraction (MVIC), showed a twofold decrease in ECC compared to CON cycling, and remained lower for 2 days. Similarly, the rate of force development was altered to a greater extent after ECC than CON cycling (Peñailillo et al., 2015b). Indeed, Peñailillo et al. (2013) found a larger diminution of force during an MVIC, and of squat jump and counter movement jump height, after a bout of ECC than CON cycling performed at the same mechanical power.

#### Perceptual Responses

At a given mechanical workload, muscle soreness was reported to be above baseline during and immediately after CON as opposed to ECC cycling, while it was higher 24 h after the end of the exercise (4.5 on a visual analog scale from 0 to 10) and 48 h (4.7) in healthy subjects (Elmer et al., 2010; Besson et al., 2013; Ward et al., 2018), and in patients with chronic heart failure (3 and 2.1 after 24 and 48 h, respectively) (Chasland et al., 2017).

At a given mechanical workload, ECC cycling resulted in a 37% lower rate of perceived exertion (Perrey et al., 2001; Elmer et al., 2010; Besson et al., 2013; Laroche et al., 2013; Lewis et al., 2018; Peñailillo et al., 2018b), and almost two times lower at a given heart rate in COPD patients (MacMillan et al., 2017). In the study of Elmer et al. (2010), participants pedaled in CON with one leg and in ECC at the same mechanical power with the contralateral leg before completing 90 s of submaximal CON cycling, at the end of which perceived exertion was reported. Muscle soreness was measured during a bilateral squat. Ratings in the two perceptual variables were similar immediately after CON and ECC cycling, while they were rated higher 24 and 48 h after the ECC bout.

At a given metabolic load, the rate of perceived exertion was found to be similar (Julian et al., 2018) or higher (Perrey et al., 2001) in ECC compared to CON cycling in obese adolescents (Julian et al., 2018).

### Adaptations to Training in ECC Cycling

Table 2 summarizes the neuromuscular and perceptual adaptations to a training period (i.e., several bouts during several weeks) in ECC in comparison to CON cycling.

**Table 2:** Comparison of adaptation to training in ECC and CON cycling.

**References**	**Sample**	**Methods**	**Main findings (in ECC compared to CON)**
Besson et al., 2013	30 CHF patients	Three 30-min sessions/week for 7 weeks at a moderate perceived effort 15 rpm in ECC vs. 60 in CON Semi-recumbent vs. standard bike	- Perceived effort and muscle pain did not differ between the two groups
Elmer et al., 2012	12 healthy individuals	60 rpm, ECC 3 times a week for 7 weeks HR from 54 to 66% of max; from 10 to 30 min Or CON cycling at the maximal intensity until the work of CON group was matched Semi-recumbent bike	- Mechanical power output was doubled at a given HR post ECC, while it remained steady during maximal CON cycling - RPE was greater and exercise duration doubled in CON - Leg stiffness and jumping power increased post ECC only
Julian et al., 2018	24 obese adolescents including 12 males and 12 females (12 CON and 12 ECC)	60–70 rpm 3 sessions of 30/week for 12 weeks 2 weeks habituation, 5 at 50% VO_2__peak_, 5 at 70% VO_2__peak_ Recumbent cycle-ergometer	- ↘ in leg fat mass and greater in leg ↗ lean mass - KE MVIC and 3-rep isokinetic ECC MVC ↗ more 3-rep isokinetic CON MVC ↗ post ECC only - Similar RPE
LaStayo et al., 2000	14 healthy males (7 CON and 6 ECC)	50–70 rpm 8 weeks 54–65% of peak heart rate Twice 15 min/week to 5 times/week for 30 min Recumbent cycle-ergometer	- Leg pain ↘ gradually vs. no ↗ post CON - Larger ↗ in MVIC - ↗ in fiber size post ECC only - Leg pain increased gradually vs. no increase in CON and was higher in average
LaStayo et al., 2008	13 healthy males (7 CON and 6 ECC)	50–70 rpm 8 weeks 54–65% of peak heart rate Twice 15 min/week to 5 times/week for 30 min Recumbent cycle-ergometer	- *VL* EMG burst during ECC ↘ 10% more compared to baseline, and its activation was 90% shorter during each pedaling cycle
Lewis et al., 2018	17 sedentary males (8 CON and 9 ECC)	Cadence not reported Twice 10–30 min/week for 8 weeks 60% CON peak power Recumbent cycle-ergometer	- No difference in KE MVIC nor in 6RM leg press - Lower perceived exertion during the sessions
MacMillan et al., 2017	15 adult males with severe chronic obstructive pulmonary disease adults	60 rpm, 10 weeks 3 times 30 min/week for 10 weeks 60–80% of CON peak power in CON, similar hear rate intensity in ECC Recumbent cycle-ergometer	- Larger ↗ in total 5-rep isokinetic work - ↗ in thigh mass and ↘ in fat thigh mass post ECC only - No global ↗ in CSA post both modalities - ↗ in type I CSA post CON - PGC-Iα and electron transport were enhanced post CON only - Lower perceived exertion during the sessions

**ECC, eccentric; CON, concentric; KE, knee extensors; MVIC, maximal voluntary isometric contraction; VL, vastus lateralis; EMG, electromyography; RM, maximal repetition; CSA, cross sectional area; rpm, revolution per minute; W, watts.**

#### Muscle Activation

LaStayo et al. (2008) tested vastus lateralis muscle activation in subjects who had participated in an 8 weeks either ECC or CON cycling training program at a fixed mechanical workload. Vastus lateralis EMG burst duration per pedaling cycle was lower in eccentrically adapted than in eccentrically naïve subjects when pedaling in ECC but similar when pedaling in CON conditions. Both groups decreased their integrated EMG per burst by similar proportions when ECC or CON cycling. Finally, eccentrically adapted subjects displayed lower integrated EMG levels per pedal cycle than their eccentrically naïve peers, both when performing ECC and CON cycling.

#### Muscle Constitution

Two studies have reported that ECC cycling induces higher muscle hypertrophy than CON cycling at a similar heart rate intensity. Compared to CON cycling, ECC cycling resulted in a 52% increase in muscle fibers cross-sectional area after 8 weeks (LaStayo et al., 2000). MacMillan et al. (2017) reported improved thigh mass (2.8%) with a loss of fat thigh mass (7%) after 10 weeks in patients with chronic obstructive pulmonary disease. In the latter study, no global cross-sectional area increase for both exercise modalities was reported, but discrepancies concerning the impact on fiber typology were documented. Co-expressor cross sectional area (fibers with more than one myosin heavy chain signal) tended to augment by 13% (not significant) as a result of ECC cycling training whereas type I fiber cross-sectional area increased by 26%, and type IIa also tended to increase (15%, but not significantly) due to CON cycling. Myofibril density remained unaffected by both cycling regimes (LaStayo et al., 2000).

Twelve weeks of ECC cycling elicited a decrease in leg fat mass (-6.5%) while CON cycling did not, and a greater increase in leg lean mass (3%) than CON at a given 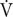
O_2_, in obese adolescents (Julian et al., 2018).

MacMillan et al. (2017) reported an enhancement of PGC-Iα (a critical transcription factor for mitochondrial biogenesis) and of the electron transport chain after CON cycling training only. In comparison, other drivers of muscular aerobic adaptation due to training (i.e., capillary density, sarcoplasmic reticulum, and mitochondria density) did not change significantly in any exercise modality (LaStayo et al., 2000).

#### Neuromuscular Performance

Gains in MVIC peak force (36%) and in total 5-rep isokinetic work during leg extension (32%) were found post-ECC cycling training but not post-CON cycling performed at the same heart rate intensity (LaStayo et al., 2000; MacMillan et al., 2017). No contraction type effects were shown for MVIC or for a 6RM leg press following training at the same mechanical workload (Lewis et al., 2018).

Julian et al. (2018) found improved knee extensor MVIC (+21%) during a 3-rep ECC isokinetic MVC (+28%) post-ECC compared to CON in obese patients. Three-rep concentric isokinetic MVC strength was significantly augmented (+16%) after ECC training only.

Elmer et al. (2012) trained a first group in ECC cycling at an intensity ranging from 54 to 65% of maximal heart rate, and another group in CON cycling who were instructed to complete the same mechanical work as the first group but “as quickly as possible”. Both groups trained three times per week for 7 weeks. Only the ECC group showed improvements in leg stiffness and jumping power.

Executing a single bout of ECC cycling elicits neuromuscular adaptations, called the “repeated bout effect” (McHugh, 2003). Indeed, A decrease in MVC, rate of force development, squat jump, and counter-movement jump performances has been observed after one bout of ECC cycling, but not after a second bout (Peñailillo et al., 2013, 2015b).

#### Perceptual Adaptation

Leg pain was reported as “very little” though decreased gradually throughout weeks of ECC cycling training while it was inexistent in the CON group at the same heart rate intensity (LaStayo et al., 2000). In their experiment described above, Elmer et al. (2012) reported a lower average rate of perceived effort during ECC than CON cycling training, despite having completed the same total mechanical work.

## Discussion

**FIGURE 2:**
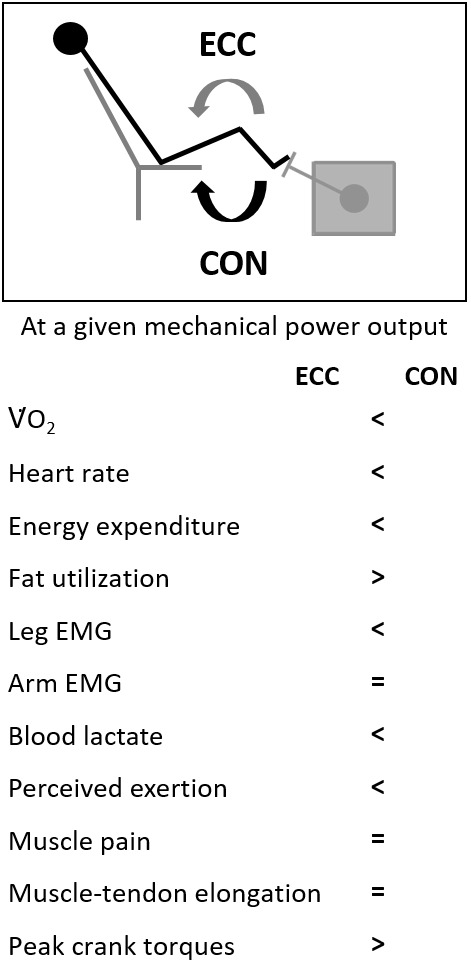
Main physiological and perceptual parameters during ECC pedaling in comparison to CON at the same mechanical workload.

The purpose of this review was to summarize recent research findings concerning neuromuscular and perceptual responses to submaximal ECC cycling compared to CON cycling. We found that acute ECC cycling differs from CON cycling by inducing lower leg muscle activation at a given mechanical workload, a larger subsequent muscle fatigue (i.e., loss of MVIC force), different perceptual responses (i.e., lower muscle pain and rate of perceived exertion), and delayed onset of muscle soreness (see Figure 2).

Chronic ECC cycling was found to be more advantageous than CON cycling in terms of muscle hypertrophy, at the same heart rate intensity or metabolic load. The extent of improvement in performance primarily involving the neuromuscular system seems essentially to depend upon the mechanical workload at which the cycling exercise is performed, which itself depends on the criterion used to match exercise intensity between the two contraction regimes. Finally, the single finding concerning the chronic perceptual responses to ECC cycling indicates a gradual decrease in leg pain compared to no reported change during CON cycling at the same heart rate intensity.

### The Singularity of Leg Muscle Command in ECC Cycling

The similar leg integrated EMG between the two modes of contraction at a given metabolic load (Lechauve et al., 2014) but lower in ECC cycling at a given mechanical workload (Bigland-Ritchie and Woods, 1976; Perrey et al., 2001; Lechauve et al., 2014; Peñailillo et al., 2017a) is coherent with intrinsic force features of ECC contraction (i.e., notably an active involvement of titin allowing a better energy yield) (Herzog, 2014). This reduced activation of leg muscles might be explained by a lower discharge rate of the motor units during ECC contractions (Pasquet et al., 2006), or by a preferential recruitment of higher-threshold motor units (Nardone et al., 1989). These peripheral neuromuscular peculiarities of ECC contractions might result from corticospinal modulations (Duchateau and Baudry, 2013). This hypothesis is coherent with studies showing lower motor evoked potentials induced by transcranial magnetic stimulation during ECC compared to CON contractions in arm flexor (Abbruzzese et al., 1994; Gruber et al., 2009) and ankle extensor muscles (Duclay et al., 2011, 2014). While the cortical excitability seems to increase (Duclay et al., 2011), spinal mechanisms, such as weaker stretch-reflexes (Duclay and Martin, 2005), might counterbalance it, leading to the aforementioned lower EMG amplitude (Gruber et al., 2009). Potential underlying mechanisms would be presynaptic -such as an inhibition of Ia afferent fibers or a diminished release of transmitters from those fibers to the motoneuron- or post-synaptic via recurrent inhibition (Duchateau and Baudry, 2013). EMG seems to be specifically modulated in eccentrically adapted subjects compared to naïve ones, during ECC cycling as well as throughout weeks of training (LaStayo et al., 2008).

Lower muscle activation corroborates the suggestion of Peñailillo et al. (2017a) that an enhanced muscular efficiency during ECC cycling is reflected by a lower oxygen utilization by the muscle due to lower ATP production.

### Perceptual Aspects

Perception of effort was found to be similar (Julian et al., 2018) or higher (Perrey et al., 2001) in ECC than CON cycling at a given metabolic load. This is coherent with the larger muscle activation of the biceps brachii in ECC compared to CON cycling at a given 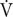
O_2_ (Lechauve et al., 2014), which leads to higher global muscle activation by adding to the activation of the main muscles involved in pedaling. Indeed, in the absence of fatigue the integrated EMG of the main muscles involved in a task can be considered as a marker of the central motor command sent to these muscles (Siemionow et al., 2000). Because a copy of the central motor command is thought to be the sensory signal generating effort perception- according to the theory of corollary discharge (Marcora et al., 2009; Pageaux et al., 2017)- the integrated EMG of the main muscles involved in a submaximal task is correlated to ratings of perceived exertion (de Morree et al., 2012). Furthermore, the larger biceps brachii integrated EMG (at the same 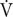
O_2_ during ECC and CON cycling) could account for a greater need for trunk stabilization due to a larger mechanical workload, which is in turn likely to limit the inspiratory capacity and could explain the compensatory increase in respiratory frequency found during ECC cycling (Lechauve et al., 2014). Interestingly, respiratory frequency was found to be strongly correlated to perceived exertion (Nicolò et al., 2016, Nicolò et al., 2017) because the corollary discharge of the central motor command triggers medullary respiratory centers, modulating respiratory frequency (Paterson, 2014).

Muscle pain was reported to be similar in the two contraction modes at a given mechanical workload (Elmer et al., 2010; Chasland et al., 2017; Ward et al., 2018), but higher at a given heart rate intensity in ECC than in CON cycling (LaStayo et al., 2000) that is at a higher mechanical workload. As at a given pedaling cadence, a higher mechanical workload must induce a larger intramuscular pressure (Gallagher et al., 2001), it therefore may provoke intensified muscle pain (Ellingson and Cook, 2013). With regards to greater levels of muscular pain observed up to 2 days after ECC than after CON cycling at the same mechanical power (Elmer et al., 2010; Chasland et al., 2017; Ward et al., 2018), the most plausible explanation resides in the muscle damage-induced inflammation provoked by ECC contractions (Fridén et al., 1983; Evans et al., 1986; Asp et al., 1995; Bruunsgaard et al., 1997; Klossner et al., 2007; Hameed et al., 2008; Flann et al., 2011). From a practical standpoint, using perception of effort as a tool to match exercise intensity between the two modes of cycling seems to be a viable manner to bypass the gap in either mechanical power output, or metabolic load (Besson et al., 2013).

### On the Origins of Muscle Damages

Larger absorption and generation of peak crank torques in ECC than CON cycling at the same average torque (Peñailillo et al., 2017a) could be responsible for greater muscle damage as evidenced by a decrease in MVIC reported following an ECC cycling bout. However, high force *per se* does not seem to provoke muscle damage – tissue elongation with high force production seems more likely to account for this (Lieber and Friden, 1993). The 8–14% greater loss of MVIC force post-ECC cycling performed with the seat of the bike positioned forward compared to backward (based on a neutral position) supports this reasoning. Indeed, the more forward the seat is the greater the muscle stretch (Peñailillo et al., 2018a). Following this rationale, the reduction in muscle fascicle elongation that occurs when performing a second ECC bout (Peñailillo et al., 2015a) might partly explain the well-known repeated bout effect (McHugh, 2003).

### Adaptations to ECC Cycling Training

ECC cycling training generates higher gains in maximal force than CON cycling does when training at the same (LaStayo et al., 2000; MacMillan et al., 2017), or a lower heart rate intensity (Elmer et al., 2012), likely due to higher training mechanical workloads. Another cause for those gains might be the development of higher torques in ECC compared to CON cycling at the same mechanical workload. In addition, ECC cycling tends toward a greater solicitation of anaerobic muscular metabolism than CON as highlighted by the enhancement of PGC-Iα and the electron transport chain along with an increased type I fibers’ cross-sectional area after CON cycling training (MacMillan et al., 2017). Nevertheless, in MacMillan’s study the metabolic load was lower in ECC than in CON cycling, which complicates the comparison of muscle aerobic-related adaptations between the two contraction modes. In fact, caution is required when using heart rate as a substitute for metabolic load measurement as its evolution seems to show a different pattern in ECC compared to CON cycling (Hesser et al., 1977; Lipski et al., 2018; Rakobowchuk et al., 2018). In a study of Mueller et al. (2009), elderly subjects performed either 12 weeks of ECC cycling or traditional resistance training. The former reduced their type IIx/II fiber ratio while the latter did not affect muscle constitution. Interestingly, the authors reported no differences in terms of maximal force gains between the two groups. These findings support the assumption that the aerobic solicitation induced by a typical submaximal ECC cycling bout is more important than that of traditional resistance training, but lower than that of CON cycling.

### Limits of This Review

Restrictive inclusion criteria were employed, especially when comparing ECC cycling to CON cycling and not to other types of exercise. The aim was to avoid confounding factors (e.g., the effect of a different movement and contraction mode on perceptual responses). Findings from different populations were presented, which included healthy, trained, and pathological participants. Individuals with divergent features are likely to respond differently to a given bout. For instance, COPD patients might perceive CON cycling to be more difficult than healthy subjects due to their respiratory limitations, but not ECC cycling at the same mechanical work rate. To our knowledge, no study compared the responses of pathological and healthy populations to ECC cycling.

Furthermore, even if most studies used recumbent cycle-ergometers, some utilized standard bicycles. Since cycling position affects the magnitude of muscle damage and soreness during the days following the training session (Peñailillo et al., 2018a), comparing findings from studies using different equipment is partially biased.

### Perspectives

Although there does not seem to be an effect of exercise modality on the improvement of MVIC when training was performed at a given mechanical workload, as ECC cycling implies inferior ratings of perceived exertion (MacMillan et al., 2017; Lewis et al., 2018) it offers a better ratio effort-benefit to patients and is likely to generate a larger compliance among them (Ekkekakis et al., 2011). However, the investigations on the topic are quite scarce and more work is needed to better develop ECC cycling in the framework of clinical settings (Pageaux et al., 2017).

Spinal and supraspinal adjustments both contribute to the modulation of the central command during ECC contractions. However, the exact mechanisms are so far unknown, and the rationales reported above arise from studies that focused on non-locomotor ECC exercise. Few studies have investigated the modulation of corticospinal mechanisms after a submaximal locomotor task (Temesi et al., 2013; Garnier et al., 2017) and none did during the actual task. Consequently, there is a need for further research upon corticospinal modulations during as well as following a locomotor ECC training. Because there are complementary methods, both stimulation-based techniques and electroencephalography should be carried-out. Moreover, data about the effects of locomotor ECC exercise on corticospinal modulation only include the study of downhill running, of which induced plyometric muscle contractions might constitute a bias when focusing on ECC. These activities are further complicated as they involve muscle groups (contracting concentrically) that are not involved in the force-production component of the activity (e.g., arms during walking or running). Therefore, ECC cycling appears to be a particularly relevant modality to investigate corticospinal modulations during a locomotor task.

Moreover, most studies employed semi-recumbent positions and backward ECC pedaling. Only Peñailillo et al. (2017b) implemented ECC cycling using forward pedaling, but did not compare it to CON cycling. Although this variant appears to be more demanding than backward pedaling, it could be of interest in order to strengthen the knee flexors and thus deserves to be explored.

## Conclusion

This review focused on two much neglected but critical aspects of ECC cycling: the neuromuscular and perceptual responses. The results indicate that the most prescribed form of ECC cycling seems to be somewhere between aerobically demanding CON cycling and muscle damaging resistance training. Very few studies have explored changes in corticospinal excitability, or the effects of bike position (i.e., recumbent or standard) and pedaling direction (backward or forward) during or upon responses to ECC cycling. Finally, the perceptual responses reported are essentially limited to perceptions of pain and effort, while complementary measures such as discomfort, pleasure, or the psychological load would be relevant in order to anticipate on patient’s compliance.

## Author Contributions

PC, RL, and DL decided of the boundaries of the review, and collected data. PC analyzed the data and drafted the manuscript. PS and RL revised the figures. All authors critically revised the manuscript and approved the final manuscript prior to submission.

## Conflict of Interest Statement

The authors declare that the research was conducted in the absence of any commercial or financial relationships that could be construed as a potential conflict of interest.
