# Corrigendum: Neuromuscular and Perceptual Responses to Sub-Maximal Eccentric Cycling

**DOI:** 10.3389/fphys.2020.00934

**Published:** 2020-08-12

**Authors:** Pierre Clos, Davy Laroche, Paul J. Stapley, Romuald Lepers

**Affiliations:** ^1^CAPS UMR1093, Institut National de la Santé et de la Recherche Médicale (INSERM), Université de Bourgogne-Franche Comté, Dijon, France; ^2^INSERM CIC 1432, Plateforme d'Investigation Technologique, University Hospital of Dijon, Dijon, France; ^3^Neural Control of Movement Group, Faculty of Science, Medicine and Health, School of Medicine, Illawarra Health and Medical Research Institute, University of Wollongong, Wollongong, NSW, Australia

**Keywords:** negative work, pedaling, perception, corticospinal, rehabilitation

In the original article, there was a mistake in Table 2 as published. We wrote that in LaStayo et al. (2000), leg pain gradually *increased*, while it gradually *decreased*. The correct statement for LaStayo et al. (2000) is leg pain gradually decreased while it gradually increased. The corrected Table 2 appears below.

**Table 2:** Comparison of adaptation to training in ECC and CON cycling.

**References**	**Sample**	**Methods**	**Main findings (in ECC compared to CON)**
Besson et al., 2013	30 CHF patients	Three 30-min sessions/week for 7 weeks at a moderate perceived effort 15 rpm in ECC vs. 60 in CON Semi-recumbent vs. standard bike	- Perceived effort and muscle pain did not differ between the two groups
Elmer et al., 2012	12 healthy individuals	60 rpm, ECC 3 times a week for 7 weeks HR from 54 to 66% of max; from 10 to 30 min Or CON cycling at the maximal intensity until the work of CON group was matched Semi-recumbent bike	- Mechanical power output was doubled at a given HR post ECC, while it remained steady during maximal CON cycling - RPE was greater and exercise duration doubled in CON - Leg stiffness and jumping power increased post ECC only
Julian et al., 2018	24 obese adolescents including 12 males and 12 females (12 CON and 12 ECC)	60–70 rpm 3 sessions of 30/week for 12 weeks 2 weeks habituation, 5 at 50% VO_2peak_, 5 at 70% VO_2peak_ Recumbent cycle-ergometer	- ↘ in leg fat mass and greater in leg ↗ lean mass - KE MVIC and 3-rep isokinetic ECC MVC ↗ more 3-rep isokinetic CON MVC ↗ post ECC only - Similar RPE
LaStayo et al., 2000	14 healthy males (7 CON and 6 ECC)	50–70 rpm 8 weeks 54– 65% of peak heart rate Twice 15 min/week to 5 times/week for 30 min Recumbent cycle-ergometer	- Leg pain ↘ gradually vs. no ↗ post CON - Larger ↗ in MVIC - ↗ in fiber size post ECC only - Leg pain increased gradually vs. no increase in CON and was higher in average
LaStayo et al., 2008	13 healthy males (7 CON and 6 ECC)	50–70 rpm 8 weeks 54–65% of peak heart rate Twice 15 min/week to 5 times/week for 30 min Recumbent cycle-ergometer	- *VL* EMG burst during ECC ↘ 10% more compared to baseline, and its activation was 90% shorter during each pedaling cycle
Lewis et al., 2018	17 sedentary males (8 CON and 9 ECC)	Cadence not reported Twice 10–30 min/week for 8 weeks 60% CON peak power Recumbent cycle-ergometer	- No difference in KE MVIC nor in 6RM leg press - Lower perceived exertion during the sessions
MacMillan et al., 2017	15 adult males with severe chronic obstructive pulmonary disease adults	60 rpm, 10 weeks 3 times 30 min/week for 10 weeks 60–80% of CON peak power in CON, similar hear rate intensity in ECC Recumbent cycle-ergometer	- Larger ↗ in total 5-rep isokinetic work - ↗ in thigh mass and ↘ in fat thigh mass post ECC only - No global ↗ in CSA post both modalities - ↗ in type I CSA post CON - PGC-Iα and electron transport were enhanced post CON only - Lower perceived exertion during the sessions

*ECC, eccentric; CON, concentric; KE, knee extensors; MVIC, maximal voluntary isometric contraction; VL, vastus lateralis; EMG, electromyography; RM, maximal repetition; CSA, cross sectional area; rpm, revolution per minute; W, watts*.

In the original article, there were two errors. We wrote that in LaStayo et al. (2000), leg pain gradually *increased*, while it gradually *decreased*.

A correction has been made to Results, Adaptations to Training in ECC Cycling, Perceptual Adaptation:

Leg pain was reported as “very little” though decreased gradually throughout weeks of ECC cycling training while it was inexistent in the CON group at the same heart rate intensity (LaStayo et al., 2000). In their experiment described above, Elmer et al. (2012) reported a lower average rate of perceived effort during ECC than CON cycling training, despite having completed the same total mechanical work.

We wrote that in LaStayo et al. (2000), leg pain gradually *increased*, while it gradually *decreased* .

A correction has been made to Discussion, Paragraph two:

Chronic ECC cycling was found to be more advantageous than CON cycling in terms of muscle hypertrophy, at the same heart rate intensity or metabolic load. The extent of improvement in performance primarily involving the neuromuscular system seems essentially to depend upon the mechanical workload at which the cycling exercise is performed, which itself depends on the criterion used to match exercise intensity between the two contraction regimes. Finally, the single finding concerning the chronic perceptual responses to ECC cycling indicates a gradual decrease in leg pain compared to no reported change during CON cycling at the same heart rate intensity.

The authors apologize for these errors and state that this does not change the scientific conclusions of the article in any way. The original article has been updated.
